# Detection of Porcine Deltacoronavirus RNA in the Upper and Lower Respiratory Tract and Biliary Fluid and the Effect of Infection on Serum Cholesterol Levels and Blood T Cell Population Frequencies in Gnotobiotic Piglets

**DOI:** 10.3390/vetsci10020117

**Published:** 2023-02-04

**Authors:** Amalie Ehlers Bedsted, Kwonil Jung, Linda J. Saif

**Affiliations:** 1 Center for Food Animal Health, Department of Animal Sciences, College of Food, Agricultural, and Environmental Sciences, The Ohio State University, Wooster, OH 44691, USA; 2 Department of Veterinary and Animal Sciences, Faculty of Health and Medical Sciences, University of Copenhagen, 1870 Frederiksberg, Denmark

**Keywords:** Porcine deltacoronavirus, bile, respiratory fluids, tissue tropism, T cells, serum cholesterol

## Abstract

**Simple Summary:**

Porcine deltacoronavirus is a newly emerged coronavirus infecting pigs. Deltacoronaviruses were previously reported mainly in birds. Porcine deltacoronavirus infects the small intestine of pigs and causes diarrhea, dehydration, and anorexia, leading to decreased body weight gain and deaths. Moreover, porcine deltacoronavirus can infect other avian or mammalian species, such as poultry and cattle, respectively. The virus has also been detected in febrile children. However, the pathogenesis in pigs or other animals is unclear. In this study, we infected germ-free piglets oronasally with porcine deltacoronavirus. We investigated the presence of the virus in various tissues, as well as differences in T cell populations (a type of immune cell), and cholesterol levels between infected and control animals. As expected, the infected piglets developed intestinal disease, but we also detected viral RNA in respiratory samples and in bile. Furthermore, the infected piglets showed trends toward lower frequencies of T cells in blood, and had higher levels of serum cholesterol compared with control animals. Our findings contribute to an understanding of porcine deltacoronavirus pathogenesis.

**Abstract:**

Porcine deltacoronavirus (PDCoV) was first identified approximately a decade ago, but much is still obscure in terms of its pathogenesis. We aimed to further characterize PDCoV infection by investigating the presence of virus in respiratory and biliary tissues or fluids; T cell population frequencies in blood; and altered serum cholesterol levels. Twelve, 6-day-old, gnotobiotic piglets were inoculated oronasally with PDCoV OH-FD22 (2.6 × 10^7^ FFU/pig). Six control piglets were not inoculated. Rectal swab (RS), nasal swab (NS), nasal wash (NW), bronchoalveolar lavage (BAL), and biliary fluid (BF) samples were collected at 2, 4, and 7 days post-inoculation (DPI) and tested for PDCoV RNA by RT-qPCR. Blood T cell populations and serum cholesterol levels were determined by flow cytometry and a colorimetric assay, respectively. Moderate to high, and low to moderate titers of PDCoV RNA were detected in RS and in NS, NW, BAL, and BF samples, respectively, of inoculated piglets. There were trends toward decreased CD4+CD8−, CD4−CD8+, and CD4+CD8+ blood T cell frequencies in inoculated piglets. Furthermore, serum cholesterol levels were increased in inoculated piglets. Overall, we found that PDCoV infection does not exclusively involve the intestine, since the respiratory and biliary systems and cholesterol metabolism also can be affected.

## 1. Introduction

Porcine deltacoronavirus (PDCoV) induces intestinal disease in pigs, but its ability to cross species barriers also enables it to infect other animals such as mice, poultry, and calves [1,2,3]. Importantly, the virus also has been identified in children with acute febrile disease [4] highlighting its zoonotic potential. The virus causes clinical disease in pigs including diarrhea, vomiting, dehydration, and mortality of around 40% in neonatal piglets [5,6,7]. Initially, PDCoV was detected in pigs in Hong Kong in 2012 [8]. In 2014 in the US, PDCoV was first identified in a porcine epidemic diarrhea virus (PEDV)-negative farm that experienced markedly elevated death rates in piglets and diarrhea in piglets and sows [7]. It subsequently caused an epidemic in US swine [9].

The main target of PDCoV infection is the intestine. PDCoV RNA and proteins have been detected in the small and large intestine by in situ hybridization and immunofluorescence (IF) staining [6]. Furthermore, viral RNA has been detected by RT-PCR in various extra-intestinal tissues including heart, lung, liver, and kidney [10,11,12,13,14,15], and PDCoV infected pigs were shown to develop mild interstitial pneumonia in a previous study [16]. Nasal or nasopharyngeal swabs have been used to assess the distribution of PDCoV in pigs [14,15], but the presence of virus in the upper respiratory tract, as determined in nasal wash (NW) samples, in the lower respiratory tract, determined using bronchoalveolar lavage (BAL), or in biliary fluids has not been investigated.

Distinct T cell populations, including CD4+CD8− (T helper cells, CD4+) and CD4−CD8+ (cytotoxic T cells, CD8+ cells), often play important roles in the host’s defense against viral infections through the development of the adaptive immune response [17]. However, there is only limited information on the T cell responses following PDCoV infection. Hence, we aimed to estimate the population frequencies of different subsets of T cells by flow cytometry analysis in the blood samples of PDCoV infected and mock control piglets.

The important role of cholesterol in cellular and/or viral membranes for coronavirus entry into cells has been reported for PDCoV [18,19], as well as for the alphacoronaviruses, PEDV, and transmissible gastroenteritis virus (TGEV) that cause clinically similar disease [20,21]. However, coronaviruses may also affect cholesterol metabolism, and it has been shown that severe acute respiratory syndrome coronavirus 2 (SARS-CoV-2) patients had lower serum concentrations of total cholesterol compared with controls [22]. In another study, it was found that PEDV led to elevated cholesterol levels in the livers of infected piglets [23]. There is no available information on serum cholesterol levels in PDCoV-infected pigs, although the close interaction between coronaviruses and cholesterol has been reviewed by Dai et al. [24].

The possibility of cross-species transmission and the current gaps in knowledge about tissue tropism and pathogenesis of PDCoV highlight the importance of expanding our knowledge of this virus. Therefore, the purpose of this study was to further characterize PDCoV infection by investigating the presence of virus in respiratory or biliary tissues or fluids, T cell populations in blood, and altered serum cholesterol levels through the early to later stages of infection. In addition to the development of clinical disease and detection of moderate to large amounts of PDCoV RNA in rectal swab (RS) samples, our study demonstrated that infected piglets had detectable levels of viral RNA in samples from the respiratory tract and in biliary fluid (BF), and trends toward lower blood T cell frequencies, as well as increased serum cholesterol compared with controls.

## 2. Materials and Methods

### 2.1. Animals

Eighteen gnotobiotic piglets from a specific pathogen-free sow were delivered by hysterectomy and kept in sterile isolator units. The piglets were fed UHT cow milk (Natrel^®^ homogenized whole milk, Agropur Inc., Grand Rapids, MI, USA) twice daily and observed frequently throughout the experiment for clinical signs.

### 2.2. Experimental Infection of Gnotobiotic Piglets with PDCoV

Six days following the hysterectomy, twelve of the piglets were inoculated with the PDCoV strain OH-FD22-P21 (cell culture passage 21 in LLC-PK1 cells) [25,26] at a titer of 1.3 × 10^7^ FFU/mL. The cell-culture adapted strain was originally isolated from a sample collected at a farm in Ohio with an outbreak of diarrhea in 2014 [25]. One mL of the virus suspension was administered orally and 0.5 mL was administered in each nostril. The remaining six piglets were not inoculated and served as controls. At 2 and 4 days post-inoculation (DPI), four inoculated piglets and two control piglets were euthanized. At 7 DPI this was repeated, but with only three piglets in the inoculated group as one piglet (no. 10) was euthanized at 5 DPI due to profound diarrhea, dehydration, and emaciation. As a result, samples from pig no. 10 were collected only at 2 and 4 DPI. The animal experiments were approved by the Institutional Animal Care and Use Committee (IACUC) of the Ohio State University.

### 2.3. Sampling

Blood for serum was drawn, and RS and nasal swab (NS) samples were collected from all gnotobiotic piglets at 2, 4, and 7 DPI. When the RS was collected, a fecal score was registered as follows: 0 (solid), 1 (pasty), 2 (semiliquid), 3 (liquid), and diarrhea was defined as scores of 2 or above [1]. Each RS was diluted 1:10, and NS 1:25 in Minimum Essential Medium (MEM). To extract serum from blood, whole blood was left to coagulate at room temperature for 30 min, centrifuged at 2,000x g for 10 min at 4 °C, and then the serum was aspirated and stored at −80 °C. At necropsy, approximately 30 mL of whole blood was collected to isolate the mononuclear cells (MNCs) using 30% (vol/vol) acid citrate dextrose (ACD) as previously reported [27]. Briefly, to prepare the ACD, we dissolved 24.5 g dextrose, 22 g sodium citrate dehydrate, and 7.3 g citric acid monohydrate in 1 L MilliQ water, adjusted the pH to 7.0–7.3, and filtered the solution. The middle part of the nasal turbinate and trachea, a piece of lung tissue from the right cranial lobe were collected and immediately fixed in 10% neutral buffered formalin.

The NW was collected by transecting the uppermost part of the trachea, injecting approximately 5 mL of MEM into each nostril and collecting the media in a sterile tube held under the trachea. The BAL was performed in the left lung by ligating the right cranial and caudal lobe bronchi. Approximately 25 mL of MEM was injected into the left lung via the trachea. The left lung was gently kneaded, and the medium was collected from the lung by pouring into a sterile tube. The BF was aspirated from the gallbladder with sterile syringes and needles.

### 2.4. RNA Extraction and RT-qPCR

RNA was extracted on a MagMax^TM^ Express machine from RS, NS, BAL, NW, and BF samples using the 5X MagMax^TM^-96 Viral RNA Isolation kit (#AMB18365, Thermo Fisher Scientific, Waltham, MA, USA) as reported previously [1,6]. After RNA extraction, the samples were stored at −80 °C. For the one-step RT-qPCR, Qiagen^®^ OneStep RT-PCR Kit (#210212, Qiagen), the forward primer 5′-ATCGACCACATGGCTCCAA-3′, reverse primer 5′-CAGCTCTTGCCCATGTAGCTT-3′, and probe FAM-CACACCAGTCGTTAAGCATGGCAAGCT-IABkFQ were used. The RT-qPCR was performed on an Eppendorf realplex^2^ Mastercycler epgradient or epgradient S machine with the software Realplex (version 2.2, Eppendorf, Germany) at 50 °C for 30 min, 95 °C for 15 min, followed by 45 cycles of 95 °C for 15 s, 56 °C for 60 s. The limit of detection (LoD) for the RT-qPCR was 10 genome equivalents (GE)/reaction, which corresponded to 3.6 log_10_ GE/mL for BAL, NW, and BF, 4.6 log_10_ GE/mL for RS, and 5.3 log_10_ GE/mL for NS as described previously [1,6]. Briefly, the LoD for RS was calculated using a standard curve and LoDs for NS, BAL, NW, and BF were calculated based on the results for RS and the dilution factor for each sample type [1,6]. For RT-qPCR, all samples were run in duplicate, and RNA extraction as well as RT-qPCR were performed with appropriate controls and repeatedly performed on outliers or unexpected results.

### 2.5. Pathology and IF Staining for the Detection of PDCoV Proteins in Respiratory Tissues

Formalin-fixed tissues (nasal turbinate, trachea, and lung) were examined histologically and tested multiple times by IF staining for PDCoV proteins, using both the hyperimmune gnotobiotic pig antiserum against PDCoV OH-FD22 strain (1:50–100) and PDCoV-specific monoclonal antibody directed against the nucleocapsid (N) protein (1:100; SDSU, mAb 55–197), and PDCoV antigen-positive intestinal tissues from a prior experiment were also tested as positive controls, as reported previously [6].

### 2.6. Isolation of Mononuclear Cells and Flow Cytometry

The MNCs were isolated from blood and resuspended in enriched Roswell Park Memorial Institute 1640 (E-RPMI) medium as reported previously [27]. After isolation, the MNCs were counted, and the viability was determined by trypan blue exclusion. Cells were stained to evaluate the frequencies of CD3+ T cell subpopulations: CD4+CD8−, CD4−CD8+, and CD4+CD8+ as described previously [28]. Flow cytometry was performed with a BD Accuri™ C6 Plus Flow Cytometer and the BD CSampler™ Plus Software (version 1.0.23.1, BD Biosciences, Franklin Lakes, NJ, USA). Frequencies of CD4+CD8−, CD4−CD8+, and CD4+CD8+ T cells were expressed as a percentage among all CD3+ cells.

### 2.7. Cholesterol Assay

Total cholesterol levels in serum were measured using a colorimetric assay kit (Total Cholesterol Assay Kit (Colorimetric), #STA-384, Cell Biolabs, Inc.). Three samples were excluded, one sample from an inoculated piglet and one sample from a control piglet from 4 DPI, and one sample from an inoculated piglet from 7 DPI, as they had the highest degree of hemolysis. Cholesterol concentrations were determined by the use of a standard curve. The assay was performed in duplicate according to the manufacturer’s instructions. Serum was diluted 1:200 in 1x assay diluent, and the plates were read by spectrophotometry in a SpectraMax 340PC384 Microplate Reader at 540 nm and the software SoftMax^®^ Pro (version 4.7.1, Molecular Devices, LLC., San Jose, CA, USA).

### 2.8. Statistics

GraphPad Prism (Version 9.4.1 for Windows, GraphPad Software, San Diego, CA, USA, www.graphpad.com, accessed on 3 September 2022) was used for statistical data analysis. For the cholesterol measurements, three separate T tests were performed on data from each day (2, 4, 7 DPI). Multiple unpaired T tests were performed on T cell frequencies for each cell type. *p* values equal to or below 0.05 were considered significant. Statistical analyses were performed with the guidance of the Section of Biostatistics at the Department of Public Health, University of Copenhagen, Denmark.

## 3. Results

### 3.1. Clinical Signs

Clinical signs of intestinal disease commenced from approximately 1 DPI and included watery or pasty yellow diarrhea and inappetence. No dyspnea, coughing, sneezing, or other signs of respiratory disease were observed. At necropsy, inoculated animals appeared more emaciated compared with controls and showed signs of dehydration, such as poor skin turgor and low blood volume. As mentioned previously, piglet no. 10 was found highly dehydrated and emaciated at 5 DPI and was euthanized based on the IACUC protocol. All inoculated piglets had fecal scores of 3 (indicative of diarrhea) and all control piglets had fecal scores of 0 (normal fecal consistency) on the scale from 0 to 3 mentioned in the Materials and Methods section (Section 2.3) at all time-points of sampling.

### 3.2. RT-qPCR

**Figure 1:**
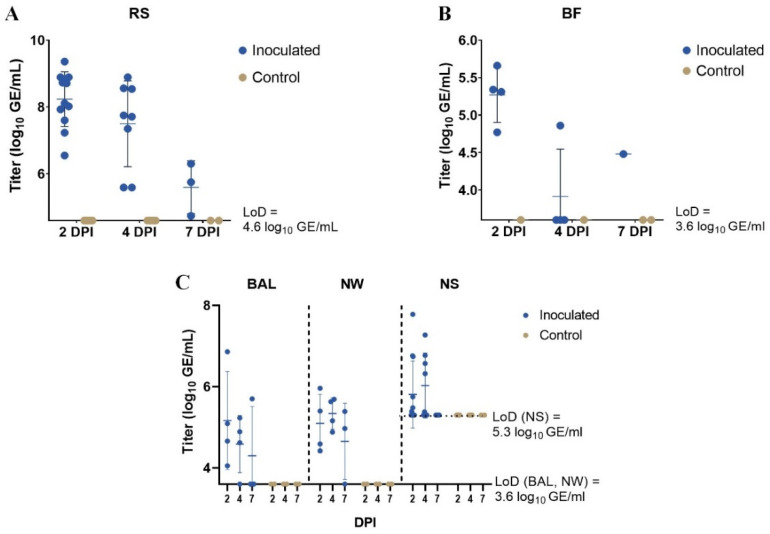
Detection of viral RNA in (**A**) rectal swab (RS), (**B**) biliary fluid (BF), (**C**) bronchoalveolar lavage (BAL), nasal wash (NW), and nasal swab (NS) samples with viral titers (log_10_ GE/mL) based on RT-qPCR results. Limit of detection (LoD) was 4.6 log_10_ genome equivalents (GE)/mL for RS, 3.6 log_10_ GE/mL for BF, BAL, and NW, and 5.3 log_10_ GE/mL for NS. The number of samples with titers above the LoD is summarized in Table 1. All negative control samples had RNA levels below the LoD. The middle line and the bars indicate the mean ± SD. The figure was created with GraphPad Prism.

The mean (±SD) PDCoV RNA titers in RS samples from inoculated piglets were 8.2 (±0.8) log_10_ GE/mL, 7.5 (±1.3) log_10_ GE/mL, and 5.6 (±0.8) log_10_ GE/mL at 2, 4, and 7 DPI, respectively (Figure 1A). All samples from inoculated piglets, i.e., 12/12, 8/8, and 3/3 at 2, 4, and 7 DPI, respectively (Table 1), had titers above the LoD of 4.6 log_10_ GE/mL. For the control piglets, all samples had levels below the LoD (Figure 1A).

Viral RNA (with titers above the LoD of 3.6 log_10_ GE/mL) was detected in 4/4, 1/4, and 1/1 BF samples from inoculated piglets at 2, 4, and 7 DPI, respectively (Table 1). In total, 13 BF samples were tested; the remaining piglets were not tested due to either early euthanasia (piglet no. 10), or too little or too dry biliary contents to allow sampling and RNA extraction. The mean (±SD) titers in BF samples from inoculated piglets were 5.3 (±0.4) log_10_ GE/mL at 2 DPI, 3.9 (±0.6) log_10_ GE/mL at 4 DPI, and 4.5 log_10_ GE/mL (only one pig) at 7 DPI (Figure 1B). The levels of viral RNA in samples from the control piglets were all below the LoD (Figure 1B).

For BAL, the mean (±SD) PDCoV RNA titers in samples from inoculated animals were 5.2 (±1.2) log_10_ GE/mL, 4.6 (±0.7) log_10_ GE/mL, and 4.3 (±1.2) log_10_ GE/mL at 2, 4, and 7 DPI, respectively (Figure 1C). Viral RNA (with titers above the LoD of 3.6 log_10_ GE/mL) was detected in 4/4, 3/4, and 1/3 BAL samples from the inoculated piglets from 2, 4, and 7 DPI, respectively (Table 1). The samples from the control piglets all had levels below the LoD (Figure 1C).

The NW samples from inoculated piglets at 2, 4, and 7 DPI had mean (±SD) PDCoV RNA titers of 5.1 (±0.7) log_10_ GE/mL, 5.3 (±0.4) log_10_ GE/mL, and 4.6 (±0.9) log_10_ GE/mL, respectively (Figure 1C). The number of samples from inoculated piglets with titers above the LoD of 3.6 log_10_ GE/mL was 4/4, 4/4, and 2/3 at 2, 4, and 7 DPI, respectively (Table 1). All NW samples from the control piglets had PDCoV RNA levels below the LoD (Figure 1C).

For NS, the mean (±SD) titers of samples from inoculated piglets were 5.8 (±0.8) log_10_ GE/mL and 6.0 (±0.8) log_10_ GE/mL at 2 and 4 DPI, respectively (Figure 1C). Viral RNA (with titers above the LoD of 5.3 log_10_ GE/mL) was detected in 6/12, 5/8, and 0/3 NS samples from inoculated pigs at 2, 4, and 7 DPI, respectively (Table 1). NS samples from inoculated animals at 7 DPI and from control piglets at all sampling days had RNA levels below the LoD (Figure 1C).

### 3.3. Pathology and IF Staining

At necropsy, distended intestines congested with watery, yellow contents were seen in all infected animals and the wall of the small intestine was thin and transparent. The control animals did not have intestinal lesions at any time point examined. No macroscopic respiratory lesions were apparent in either group. Light microscopy analysis revealed that none of the PDCoV-inoculated or control piglets had major histological changes in the nasal turbinate, trachea, and lung tissues tested. No PDCoV antigen-positive cells were evident by our IF staining conditions in the nasal turbinate, trachea, and lung tissues of inoculated or control piglets, although PDCoV antigen-positive cells were detected in intestinal tissues of the infected piglets.

### 3.4. Blood T Cell Population Frequencies as Determined by Flow Cytometry

T cell population frequencies in the blood of inoculated and control piglets are summarized in Table 2.

**Figure 2:**
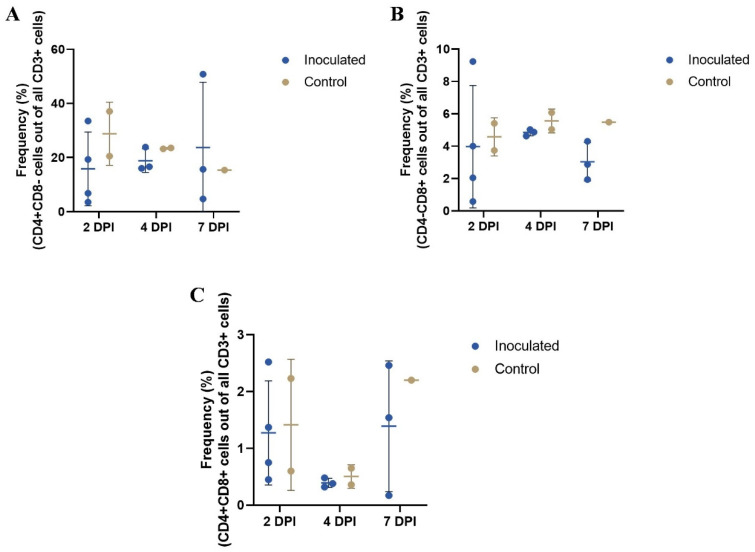
T cell frequencies in blood samples from the inoculated and the control group. A, B, and C show the frequencies of T cells at each day: CD4+CD8− (**A**), CD4−CD8+ (**B**), and CD4+CD8+ (**C**). The number of samples from inoculated and control animals was 4, 3, and 3, and 2, 2, and 1 at 2, 4, and 7 DPI, respectively. In the multiple unpaired T-tests performed on each cell type, there was no significant difference between the groups at 2, 4, or 7 DPI. The middle line and the bars indicate the mean ± SD. The figure was created with GraphPad Prism.

The gating strategies for the CD4+CD8−, CD4−CD8+, and CD4+CD8+ T cells are illustrated in the supplementary material (see Supplementary Materials, Figure S1). The number of samples from inoculated animals was 4, 3, and 3 and from control animals, the number of samples was 2, 2, and 1 at 2, 4, and 7 DPI, respectively. In the multiple unpaired T tests performed on each cell type (CD4+CD8−, CD4−CD8+, CD4+CD8+), no significant differences were found between inoculated and control groups at 2, 4, or 7 DPI (see Supplementary Materials, Table S1, for *p* values). However, there was a trend toward lower T cell population frequencies in the blood of inoculated piglets compared with control piglets on all sampling days (Figure 2).

### 3.5. Cholesterol Assay

**Figure 3:**
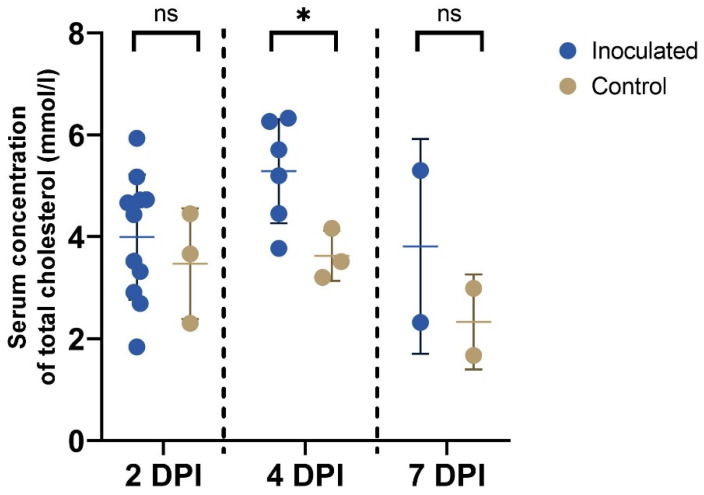
Concentration of total cholesterol (mmol/l) in serum samples. The number of tested samples (non-hemolyzed) was 11, 6, and 2 for inoculated animals and 3, 3, and 2 for control animals at 2, 4, and 7 DPI, respectively. Three separate T tests were performed, one for each day. There was a significant difference between inoculated and control animals at 4 DPI (*p* = 0.035, marked with *), but not at 2 or 7 DPI (marked with ns). The middle line and the bars indicate the mean ± SD. The figure was created with GraphPad Prism.

The mean (±SD) values of total cholesterol in serum from inoculated piglets were 4.0 (±1.2) mmol/l, 5.3 (±1.0) mmol/l, and 3.8 (±2.1) mmol/l at 2, 4, and 7 DPI, respectively. For control piglets, the mean concentrations were 3.5 (±1.1) mmol/l, 3.6 (±0.5) mmol/l, and 2.3 (±0.9) mmol/l. There was a trend toward higher mean total cholesterol levels in piglets infected with PDCoV on all sampling days, although there were only two piglets in each group at 7 DPI. Based on the T test performed for each day, there was a significantly higher total cholesterol level in inoculated compared with control piglets at 4 DPI (*p* = 0.035, 95% confidence interval (CI) −3.2 to −0.2), but not at 2 DPI (*p* = 0.52, 95% CI −2.2 to 1.2) and 7 DPI (*p* = 0.46, 95% CI −8.5 to 5.5) (Figure 3).

## 4. Discussion

PDCoV is a recently identified coronavirus in pigs, first reported in Hong Kong in 2012 [8]. Similar to TGEV and PEDV, it causes increased morbidity and mortality, especially in piglets. In the present study, we aimed to further investigate PDCoV tissue tropism and pathogenesis. We found that PDCoV infection of gnotobiotic piglets led to enteric disease with watery diarrhea and severe gross lesions such as thin-walled, distended intestines, and shedding of moderate to high levels of viral RNA in feces at 2–7 DPI. In addition to the enteric disease observed, PDCoV infection was characterized by 1) detection of viral RNA in BF; 2) detection of viral RNA in various samples from the upper and lower respiratory tract: BAL, NW, and NS; 3) apparent trends toward decreased CD4+CD8− (T helper cells), CD4−CD8+ (cytotoxic T cells), and CD4+CD8+ (double-positive T cells) compared with non-inoculated control piglets; and 4) elevated serum cholesterol levels compared with the control piglets.

In our study, a short incubation time was demonstrated by the commencement of clinical signs at 1 DPI. The RT-qPCR data on RS samples showed that shedding of viral RNA in feces was highest at each time point of all sample types tested. Fecal shedding of viral RNA peaked at 2 DPI, then decreased but was still detectable at 7 DPI. Other studies have reported that clinical signs begin at 1–4 DPI [6,10,12,13,14,26], and that peak fecal shedding typically occurs concurrently (between 1–4 DPI) [6,12,13,14,26]. However, this may be affected by several factors such as duration of the experiment, sampling time-points, and viral inoculum titer and strain.

The number of samples tested in each analysis can be found in the supplementary material (see Supplementary Material, Table S2). At all sampling days, at least one BF sample from the inoculated piglets had detectable levels of PDCoV RNA. Previously, coronavirus RNA has been detected by RT-PCR in BF from a SARS-CoV-2 patient [29] and in liver tissue of pigs after PDCoV infection [10,11,12,13]. These observations suggest that after the initial infection and replication in the intestine, PDCoV may be transported via the hepatic portal system to the liver and transferred into the BF. The BF is secreted by hepatocytes and biliary tract epithelial cells, known as cholangiocytes [30]. Whether PDCoV can infect hepatic and biliary tract cells has, to our knowledge, not yet been investigated. Kong et al. recently found that the bile acids chenodeoxycholic acid (CDCA) and lithocholic acid (LCA) inhibited PDCoV replication in vitro [31]. This suggests that PDCoV may not replicate efficiently in the hepatic and biliary system, but it is nevertheless likely able to use the enterohepatic circulation to reach the liver and then re-enter the small intestine via the common biliary duct. Likewise, PDCoV present in the hepatic system may enter the systemic circulation via the hepatic vein and generate a transient viremia. PDCoV RNA has been found repeatedly, but transiently, by RT-PCR in the serum or blood from infected pigs, as reported in previous studies [10,14,16,26].

In addition to the detection in BF, low to moderate titers of PDCoV RNA were frequently detected in the BAL, NS, and NW samples from most inoculated piglets. Similarly, previous studies reported that PDCoV RNA was detected by RT-PCR in the respiratory tract as well as other extra-intestinal organs [10,11,12,13,14,15]. Moreover, PDCoV infection has led to histopathological lesions in the lungs of infected pigs in a previous study [16]. However, PDCoV antigens or RNA were not detected in the respiratory tissues by immunohistochemistry or in situ hybridization [6,10]. Consistent with the previous studies [6,10], we failed to find viral antigen in respiratory tissues using IF staining, in which two different primary antibodies against PDCoV at different concentrations were employed to increase the possibility of viral antigen detection. However, the positive control PDCoV-infected intestinal tissues showed antigen-positive cells by IF staining. There are several possibilities for these negative results: (1) difficulties with in situ identification of low amounts of viral antigen using conventional IF staining procedures; and (2) transient, limited, or abortive infectivity of PDCoV in the upper and lower respiratory tract of pigs. Only the middle part of the nasal turbinate and trachea and the right cranial lung lobe were tested for viral antigen by IF staining in our study. Thus, various other respiratory tissues collected at multiple different locations and times are needed for a more comprehensive investigation. In the present study, we inoculated the piglets both intranasally and orally, whereas previous studies used oral or orogastric inoculation [6,10,12,13,16,26]. The intranasal inoculation might have increased the presence of PDCoV RNA in the respiratory tract. However, some of the previous studies using oral or orogastric inoculation also detected PDCoV RNA in respiratory tissues [10,12,13,16]. In previous studies, potential alterations in the enteropathogenicity as well as nucleotide and amino acid changes in the S gene of PDCoV OH-FD22 following serial passages in LLC-PK cells have been investigated. Even though the enteropathogenicity was similar for the original OH-FD22 compared to the virus passaged 20 and 40 times in LLC-PK cells, it is not known how the observed nucleotide and amino acid changes in the cell-passaged virus affect the strain’s affinity for the respiratory system [25,26]. Collectively, our observations indicate a mild respiratory infection by PDCoV, but with limited capacity to replicate in the upper and lower respiratory tract and with no observed signs of respiratory disease. The pathogenesis and tissue tropism of PDCoV may be more complex than only an infection of the intestine and may also be influenced by the pig microbiota that is absent in the gnotobiotic pigs in our study. Additionally, our study reconfirmed that PDCoV infects mainly the intestine and produces severe diarrhea and sometimes deaths in neonatal pigs.

As a part of the host’s immune defense, viral infections lead to activation of the adaptive immunity system, of which the T cells are an important component. In the present study, we investigated the frequency of CD4+CD8−, CD4−CD8+, and CD4+CD8+ T cell subpopulations in the CD3+ T cells from the blood of inoculated and non-inoculated control piglets. In our study, both the inoculated and control group piglets had higher mean frequencies of CD4+CD8− T cells compared with CD4−CD8+ T cells in the blood at each sampling day. Higher CD4+ T cell compared with CD8+ T cell frequencies in blood have been found in a study of rotavirus infection of gnotobiotic piglets [28], and in PEDV infection of conventional piglets [32].

In our study, there were trends toward lower frequencies of blood CD4+CD8−, CD4−CD8+, and CD4+CD8+ T cells in inoculated compared with control piglets. The relatively lower frequencies of both blood CD4+CD8− and CD4−CD8+ T cells in PDCoV-infected piglets during 2–7 DPI is partially in agreement with previous findings of PEDV-infected suckling piglets in a study by Annamalai et al. [33]. In their study, they found that blood CD4+CD8− and CD4−CD8+ T cells of infected suckling piglets were significantly reduced at 1 DPI, but not at 5 DPI compared with uninfected piglets. Based on these observations, PEDV-infected suckling piglets appeared to have transient lymphopenia or leukopenia during the early stages of infection [33]. Lymphopenia is not uncommon in viral infections [34], and although not examined in our study, our current observations could potentially be ascribed to the activity of anti-inflammatory regulatory T cells in neonates that suppress or delay the other adaptive T cell responses [35,36].

Enzymes involved in cholesterol and fatty acid metabolism may be differentially expressed in infected animals, as has been shown in PEDV and PDCoV infections [23,37]. Some of these enzymes are also involved in inflammatory pathways, such as fatty acid-binding protein 1 (FABP1) [37]. In the present study, we found trends toward higher total cholesterol levels in the sera of PDCoV-inoculated piglets at 2–7 DPI compared with control piglets. Indeed, at 4 DPI, PDCoV-inoculated piglets had significantly higher total serum cholesterol levels compared with control piglets (*p* = 0.035). Similar to our findings, the study by Liu et al. showed that PEDV infection led to increased levels of cholesterol and bile acids in liver tissue [23]. On the other hand, our findings are contrary to previous studies performed on SARS-CoV-2 patients [22,38]. In those studies, SARS-CoV-2 patients had lower total cholesterol levels than non-infected individuals, and low total cholesterol was associated with more severe SARS-CoV-2 disease [22,38]. It can be speculated about whether this difference in the effect of coronavirus infection on cholesterol levels is linked to the differences in tissue tropisms of SARS-CoV-2 (respiratory) and PDCoV (enteric). Indeed, dehydration and lipolysis as a consequence of diarrhea following PDCoV infection could influence the serum cholesterol levels [39,40], and the inoculated piglets in our study did show signs of dehydration such as poor skin turgor and low blood volume. To further pursue this question, an examination of serum cholesterol levels in SARS-CoV-2 patients who mainly present with diarrhea or gastroenteritis could be informative. Moreover, additional studies are needed to investigate the mechanisms by which PDCoV affects cholesterol levels in serum, and whether it is a primary effect due to the virus, or a secondary effect following clinical disease such as dehydration, or indeed a combination of both.

Cholesterol plays an essential role in PDCoV entry into cells [19,41]. It could therefore be expected that statins, inhibitors of 3-hydroxy-3-methylglutaryl coenzyme A (HMG CoA) reductase, a key enzyme in cholesterol synthesis, reduce serum or cellular cholesterol levels, possibly inhibiting replication of coronaviruses, including PDCoV. Recently, much research has been performed on the effects of cholesterol-lowering statin medication on SARS-CoV-2 infection [24]. However, a previous study also found that in the case of a human enteric virus, human norovirus, statins could enhance virus infection in a gnotobiotic pig model [42]. Statins can be used to treat hypercholesteremic patients, and as the authors of a study on serum cholesterol and SARS-CoV-2 note, the different statin treatment regimens directed towards the SARS-CoV-2 patients’ co-morbidities may have lowered the serum cholesterol levels of these patients [38]. Whereas some viruses, such as SARS-CoV-2, induced hypocholesterolemia, PDCoV infection resulted in increased serum cholesterol levels, as observed in the present study. Similar to other coronaviruses, PDCoV infection may, directly or indirectly, affect cholesterol and fatty acid metabolism, which is tightly connected to inflammatory pathways [24]. However, the reasons for the increased total serum cholesterol in PDCoV infection are unclear.

## 5. Conclusions

In this study, we have investigated three new aspects of PDCoV pathogenesis: tissue tropism, T cell responses, and serum cholesterol levels in an experimental infection study of gnotobiotic piglets. We successfully established an enteric infection supported by clinical signs and detection of viral RNA in RS, as well as in BF samples. Furthermore, we detected low to moderate PDCoV RNA titers in BAL, NW, and NS samples from inoculated animals. This could potentially indicate a mild respiratory infection of PDCoV, but with limited productive replication in the respiratory tract, indicated by the lack of viral antigens in respiratory tissues as determined by IF staining. The inoculated piglets showed trends toward lower mean frequencies of blood CD4+CD8−, CD4−CD8+, and CD4+CD8+ T cell subpopulations compared with the control piglets at all sampling times, and significantly higher total cholesterol levels in serum compared with the non-inoculated piglets at 4 DPI. PEDV and PDCoV share a similar tissue or cellular tropism in the small and large intestine [6,10,11,12,13,14,16,26], and the former virus (PEDV) was reported to disseminate from an initial transient nasal epithelium infection to cause enteric disease [43]. Therefore, further studies are needed to investigate if PDCoV also employs the upper respiratory tract prior to causing enteric disease and transmitting the virus to susceptible pigs or other animal species.

## Figures and Tables

**Table 1:** Viral RNA detection in rectal swab (RS), biliary fluid (BF), bronchoalveolar lavage (BAL), nasal wash (NW), and nasal swab (NS) samples from inoculated and control piglets at 2, 4, and 7 days post-inoculation (DPI). Number of positive samples by RT-qPCR (i.e., with titers above the limit of detection (LoD)) of all tested within a sample type.

	DPI	RS	BF	BAL	NW	NS
Inoculated piglets	2	12/12	4/4	4/4	4/4	6/12
	4	8/8	1/4	3/4	4/4	5/8
	7	3/3	1/1	1/3	2/3	0/3
Control piglets	2	0/6	0/1	0/2	0/2	0/6
	4	0/4	0/1	0/2	0/2	0/4
	7	0/2	0/2	0/2	0/2	0/2

**Table 2:** Mean frequencies (±SD) of each T cell subpopulation of all CD3+ cells in the blood of inoculated (I) and control (C) piglets. * Only one piglet.

	CD4+CD8−		CD4−CD8+		CD4+CD8+	
	I	C	I	C	I	C
2 DPI	15.8% (±13.6%)	28.8% (±11.7%)	4.0% (±3.8%)	4.6% (±1.2%)	1.3% (±0.9%)	1.4% (±1.2%)
4 DPI	18.9% (±4.4%)	23.4% (±0.3%)	4.8% (±0.2%)	5.6% (±0.7%)	0.4% (±0.1%)	0.5% (±0.2%)
7 DPI	23.7% (±24.1%)	15.4% *	3.0% (±1.2%)	5.5% *	1.4% (±1.2%)	2.2% *

## Data Availability

The data set analyzed for the current study is available from the corresponding authors upon request.

## Supplementary Materials

The following supporting information can be downloaded at https://www.mdpi.com/article/10.3390/vetsci10020117/s1, Figure S1: Gating strategies for T cell population frequencies in blood samples from inoculated and control animals. Table S1: *p* values for statistical tests on data for T cell population frequencies. Table S2: Numbers of piglets (or samples) tested in each analysis at 2, 4, and 7 DPI.
