# Supplementary material for: Detection of Porcine Deltacoronavirus RNA in the Upper and Lower Respiratory Tract and Biliary Fluid and the Effect of Infection on Serum Cholesterol Levels and Blood T Cell Population Frequencies in Gnotobiotic Piglets
Source: Vet Sci. 2023 Feb 4;10(2):117. doi: 10.3390/vetsci10020117 (PMC9962660; doi:10.3390/vetsci10020117)
Supplement: Supplementary file 1 [file vetsci-10-00117-s001.zip › Supplementary Materials Figure S1.pdf]

Figure S1: Gating strategies for T cell population frequencies in blood samples from inoculated and control animals

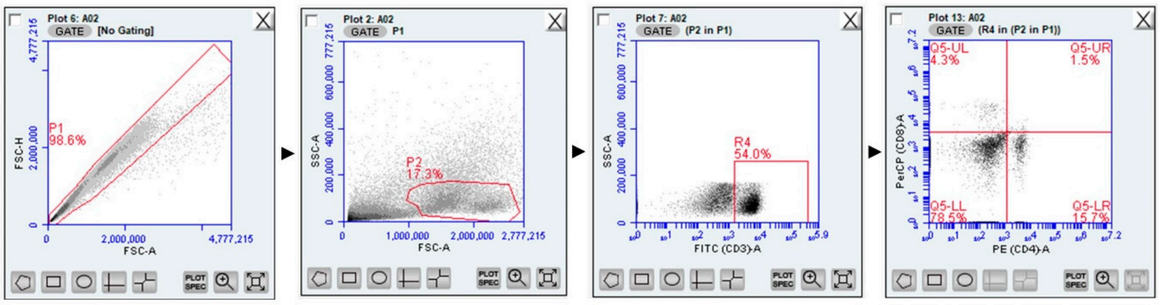

Figure S1: Gating strategies for estimating frequencies of T cells, here shown for one sample as an example. CD3+ cells were stained with FITC, CD4+ cells with PE, and CD8+ cells with SPRD detected with the PerCP filter. R4 corresponds to the population of CD3+ cells (plot 7). In the plot to the right (plot 13), the lower right (LR) quadrant indicates the frequency of CD4+CD8− cells (T helper cells, 15.7%), the upper left (UL) quadrant indicates the frequency of CD4−CD8+ cells (cytotoxic T cells, 4.3%), and the upper right (UR) quadrant indicates the frequency of CD4+CD8+ (double-positive T cells, 1.5%).
