# Supplementary material for: Detection of Porcine Deltacoronavirus RNA in the Upper and Lower Respiratory Tract and Biliary Fluid and the Effect of Infection on Serum Cholesterol Levels and Blood T Cell Population Frequencies in Gnotobiotic Piglets
Source: Vet Sci. 2023 Feb 4;10(2):117. doi: 10.3390/vetsci10020117 (PMC9962660; doi:10.3390/vetsci10020117)
Supplement: Supplementary file 1 [file vetsci-10-00117-s001.zip › Supplementary Materials Table S1.pdf]

Table S1: ***p*** **values for statistical tests on data for T cell population frequencies**

	2 DPI	4 DPI	7 DPI
CD4+CD8−	0.59 (11.41)	0.59 (3.27)	0.79 (27.80)
CD4−CD8+	0.84 (2.88)	0.45 (0.42)	0.45 (1.37)
CD4+CD8+	0.87 (0.85)	0.82 (0.12)	0.84 (1.33)

Table S1. P values obtained by multiple unpaired T tests on data for T cell population frequencies when testing for a difference between the inoculated group and the control group. The number in parenthesis is the SE of the difference between the groups. The P value is adjusted with the Holm-Šídák method for multiple tests.
