# Supplementary material for: Detection of Porcine Deltacoronavirus RNA in the Upper and Lower Respiratory Tract and Biliary Fluid and the Effect of Infection on Serum Cholesterol Levels and Blood T Cell Population Frequencies in Gnotobiotic Piglets
Source: Vet Sci. 2023 Feb 4;10(2):117. doi: 10.3390/vetsci10020117 (PMC9962660; doi:10.3390/vetsci10020117)
Supplement: Supplementary file 1 [file vetsci-10-00117-s001.zip › Supplementary Materials Table S2.pdf]

Table S2: Numbers of piglets (or samples) tested in each analysis at 2, 4, and 7 DPI

Analysis	Inoculated piglets			Control piglets		
	2 DPI	4 DPI	7 DPI	2 DPI	4 DPI	7 DPI
	Number of piglets (or samples) tested			Number of piglets (or samples) tested		
RT-qPCR: RS	12	8	3	6	4	2
RT-qPCR: BF	4	4	1	1	1	2
RT-qPCR: BAL	4	4	3	2	2	2
RT-qPCR: NW	4	4	3	2	2	2
RT-qPCR: NS	12	8	3	6	4	2
Flow cytometry: T cell population frequencies	4	3	3	2	2	1
Colorimetric assay: Serum total cholesterol	11	6	2	3	3	2

Table S2: Numbers of piglets (or samples) tested in RT-qPCR, flow cytometry, and colorimetric analyses. For RT-qPCR, rectal swab (RS), biliary fluid (BF), bronchoalveolar lavage (BAL), nasal wash (NW), and nasal swab (NS) samples were tested. T cell population frequencies were measured in blood samples of piglets.
